# Dataset on contaminated steel/rubber contact: Effect of surface texture on friction coefficient

**DOI:** 10.1016/j.dib.2025.111656

**Published:** 2025-05-19

**Authors:** Bilel Jebali, Manuela Gennesseaux, Malal Kane

**Affiliations:** Université Gustave Eiffel, Campus de Nantes, AME/EASE, Allée des ponts et chaussées, 44340 Bouguenais, France

**Keywords:** Tire/rail contact, Grooved and shot-peened samples, Texture parameters, Pin-on-disc test, Wet contact, Grease-wetted contact

## Abstract

This data article presents an experimental dataset on the friction behaviour of steel/rubber contact under contaminated conditions, obtained using a pin-on-disc tribometer. The dataset includes time-resolved coefficient of friction (CoF) values recorded over 420 seconds per test for four steel samples with distinct surface textures (shot-peened and grooved). During testing, the disc rotated at a constant speed of 60 rpm. Three contamination protocols were applied: single wetting, periodic wetting, and grease application with periodic wetting. Additionally, the dataset provides detailed surface texture parameters derived from 3D surface mapping, and calculated following ISO 25178 standards. These data can be used to analyse the influence of surface texture on frictional behaviour under degraded contact conditions, validate numerical friction models, and support the development of engineered surfaces for optimized tyre-rail interactions. The dataset is available in CSV format and can be accessed using any text editor or data analysis software. This data article is related to the research article *“Tyre/Rail Contact: An Experimental Investigation on Contaminated Contact Cases”*, where the full experimental methodology is described.

Specifications Table

**Table:** 

Subject	Engineering & Materials science
Specific subject area	Tribology, transportation engineering
Type of data	CSV files
Data collection	The data were collected from friction tests performed on four steel samples with distinct surface textures (shot-peened and grooved) using a pin-on-disc tribometer at room temperature. The disc rotated at a constant speed of 60 rpm under a normal load of 24 N. The coefficient of friction (CoF) was recorded continuously over 420 s per test. The dataset includes multiple measured variables, including normal force, vertical displacement, and friction force. The surface texture parameters were measured using a 3D optical profilometer and processed with MountainsMap Expert 9 software. The surface planarity was corrected using the Least Square Plane method. Missing data points were interpolated using a soft shape reconstruction, derived from neighbouring data. A resampling procedure was applied to achieve a resolution step of 3.47 µm. No additional filtering was applied, and the software configuration was maintained in its default state for this technical evaluation.
Data source location	EASE laboratory at Gustave Eiffel university, Nantes campus.
Data accessibility	JEBALI, B. (2025). Dataset on contaminated steel/rubber contact: Effect of surface texture on friction coefficient [Data set]. Zenodo. 10.5281/zenodo.14959103
Related research article	JEBALI, Bilel and Gennesseaux, Manuela and KANE, Malal, Tire/Rail Contact: An Experimental Investigation on Contaminated Contact Cases. Available at SSRN: https://ssrn.com/abstract=5047142 or 10.2139/ssrn.5047142

## 1 Value of the Data

• The dataset offers valuable insights into the frictional behaviour of steel-rubber contacts under contaminated conditions, which is particularly relevant for applications such as tyre-rail interaction and other tribological studies involving elastomer-metal interfaces.
• Researchers and engineers working in the fields of railway transport, automotive systems, or surface engineering can use this dataset to evaluate the influence of surface texture on friction performance under degraded or contaminated environments.
• Dataset on contaminated steel/rubber contact: Effect of surface texture on friction coefficient.
• This dataset also serves as a solid foundation for the development of predictive models of frictional behavior, particularly those based on surface texture parameters, using machine learning and other data-driven techniques.

## 2 Background

The motivation for compiling this dataset is to contribute to a better understanding of tyre-rail interactions under adverse conditions, particularly regarding the role of surface texture in frictional behaviour. In the context of innovative transportation solutions, adapting automobiles to operate on railway tracks offers promising opportunities for multimodal transport. However, the success of such a transition relies on the ability to control and optimize the frictional performance between tyres and rails, especially under degraded contact conditions.

Rain, humidity, and contaminants like motor oil can alter tyre-rail friction, reducing traction and raising safety concerns. This dataset examines steel/rubber friction under controlled contamination, providing valuable insights into the mechanisms influencing frictional behaviour. It allows researchers to assess how surface texture influences friction oscillations over time and under varying conditions. Additionally, it provides a solid basis for optimizing surface engineering to enhance friction, prevent slippage, and ensure safer and more efficient railway travel.

This data article is related to the original research article “Tyre/Rail Contact: An Experimental Investigation on Contaminated Contact Cases” [1], which details the experimental methodology and analysis. By sharing these data, this article supports research and innovation in tribology, railway safety, and transportation engineering.

## 3 Data Description

The dataset contains surface texture parameters and friction measurements for four steel samples (BL5, GL2, ES3, ES5) with different surface textures (shot-peened and grooved). Specimens are S235 steel commonly used in railways and prepared in accordance with the EX 10025 standard [2]. The implementation of surface modification process is specifically aimed at generating markedly different surface topographies, thereby enabling a controlled investigation of the influence of distinct texture characteristics on frictional performance. Following these treatments, the surface textures data were characterized using a 3D optical profilometer, and texture parameters were calculated using advanced surface metrology techniques according to ISO25178 [3].

Friction measurements were collected using a pin-on-disc tribometer under controlled conditions, with a constant disc rotation speed of 60 rpm, a normal load of 24 N, and a test duration of 420 s per experiment.

The dataset is organized into two main folders:

### 3.1 Surface_texture_parameters_ISO25178

This folder contains surface texture parameters for each sample, calculated following ISO 25178 standards, with a resolution step of 3.48 µm. The dataset provides all texture parameters defined by the standard. Each sample has a corresponding CSV file containing its texture parameters:

• BL5_texture_parameter_ISO25178.csv
• GL2_texture_parameter_ISO25178.csv
• ES3_texture_parameter_ISO25178.csv
• ES5_texture_parameter_ISO25178.csv

Additionally, the file includes a PDF document providing further details on each surface, including the Abbott curve, fractal analysis, DSP analysis, and other relevant data. The document also includes 3D surface representations, pseudo-colour views, and various statistical distributions to facilitate a deeper understanding of the sample's topography.

### 3.2 COF_measurements_pin_on_disk

This folder contains friction measurements obtained from tribometer tests under three different contamination protocols:

• Protocol 1**:** Single wetting at the start of the test.
• Protocol 2**:** Periodic wetting every 2 min.
• Protocol 3**:** Grease application with periodic wetting every 2 min.

Each subfolder includes CSV files named according to the sample and test protocol, following this structure:

• GL2-Protocol_1_wet_ones-F=24N-420s.csv
• GL2-Protocol_2_wet_2min-F=24N-420s.csv
• GL2-Protocol_3_wet_2min_greased-F=24N-420s.csv

Similar files exist for BL5, ES3, and ES5 samples. Each CSV file contains multiple measured variables:

• Fx (Normal force)
• Fz (Vertical force)
• Z, Y, X (Displacement)
• V1, V2, V3 (Velocity components)
• T (Time in seconds)
• Ff (Friction force)
• COF (Coefficient of friction)

## 4 Experimental Design, Materials and Methods

### 4.1 Steel disc samples

Four circular steel samples (BL5, GL2, ES3, ES5, 80 mm in diameter) were used to obtain texture parameters dataset. See Table 1.

**Table 1:** The samples, their surface cartography, surface profiles.

BL5 and GL2 underwent shot peening, while ES3 and ES5 featured both grooves and shot peening, allowing for the analysis of different surface morphologies. Grooves introduce additional texture variations, potentially influencing tribological properties. Varying shot peening levels further modify surface roughness and topography.

The measurements of texture parameters were taken on a redressed plane (LS) with non-measured points filled. The scanning parameters were:

• X-axis: length 20.55 mm, 5894 points, step size 3.487 µm
• Y-axis: length 18.66 mm, 5351 points, step size 3.487 µm

Table 2 presents a representative example of the surface texture parameters calculated according to ISO 25178, including height parameters (Sq, Sv, Sz) and feature parameters (Spd, Svd) for the four tested samples.

**Table 2:** A representative set of surface texture parameters (ISO 25178).

	Parameter’s category				
	Height parameters			Feature parameters	
	Sq (mm)	Sv (mm)	Sz (mm)	Spd (1/mm²)	Svd (1/mm²)
BL 5	0.02095	0.06528	0.2232	7.862	7.4358
GL 2	0.03642	0.11301	0.2444	8.944	10.418
ES 3	0.09304	0.5030	1.028	0.592	0.5948
ES 5	0.3361	0.948	1.576	0.162	0.1435

## 5 Friction Measurements

### 5.1 Used rubber

**Fig. 1:**
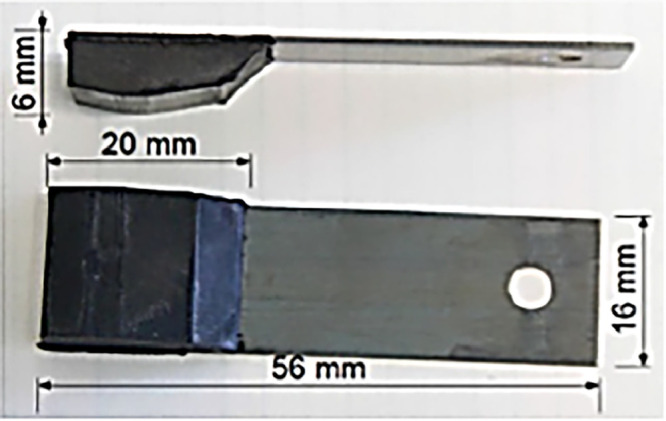
The rubber pad used in Cof measurement.

The rubber used in the tests is identical to the pad utilized in the Dynamic Friction Tester (DFT) measuring instrument, as specified in ASTM E1911 [4]. This rubber is a widely recognized standard in friction studies and its behaviour on road surfaces. The pad dimensions are 20 mm in length, 16 mm in width, and 6 mm in height, conforming to the standards for this type of equipment. The shape and geometry of the pad used for the data acquisition are shown in Fig. 1.

### 5.2 Coefficient of friction measurement

The coefficient of friction data was acquired using a tribometer configured for a pin-on-disc test, a widely used method in tribological studies. The primary goal was to record the temporal evolution of CoF under controlled conditions.

During data acquisition, the rubber pad was positioned at a radius of 23 mm from the disk center. Then a normal load of 24 N was applied while maintaining a constant rotating speed of 60 rpm. The implemented procedure allows for replication of a slip rate of 1 % at a rolling speed of approximately 60 km/h. The acting contact pressure is 0.25 MPa, corresponding to the typical contact pressure between the road and the tire of a light vehicle weighing around 3.5 tonnes. The test lasted 420 s, with friction forces continuously recorded at a sampling rate of one measurement per second, ensuring high-resolution tracking of friction variations.

**Fig. 2:**
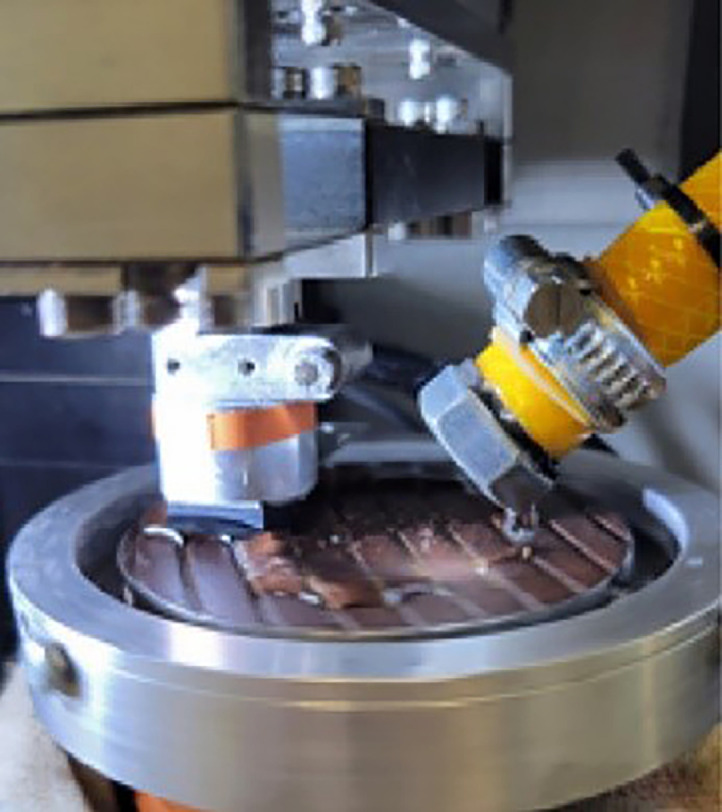
The tribometer, the positioning of the rubber pad and the sample.

The data collection process consisted of two phases: a 10-second static loading phase to ensure proper pad-disc contact, followed by a dynamic phase where the disc was set in motion at the predefined speed. The recorded data provides detailed insights into friction behaviour under conditions replicating a 0.3 MPa contact pressure and a 1% slip rate, corresponding to a rolling speed of 60 km/h. The experimental setup is illustrated in Fig. 2.

### 5.3 Applied protocols

**Fig. 3:**
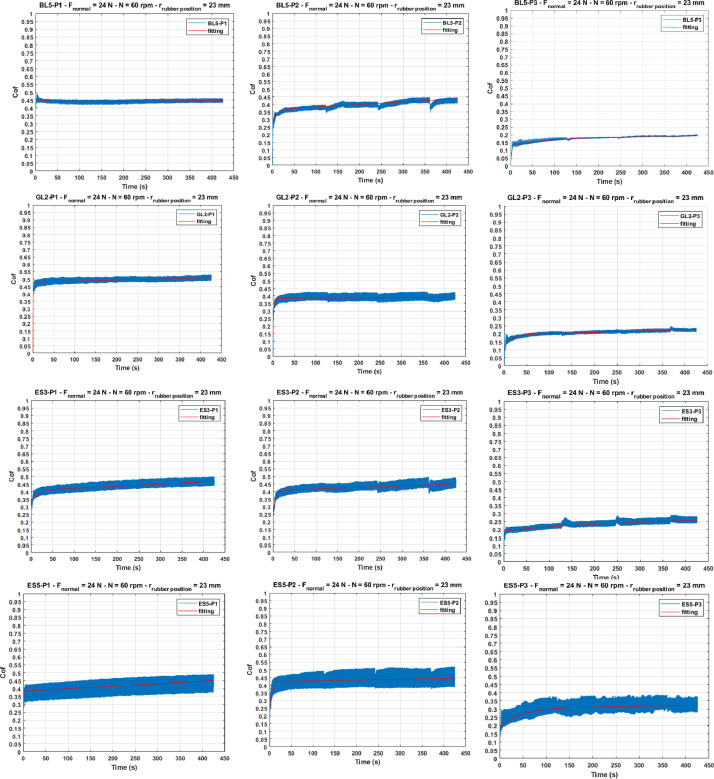
The evolution of the coefficient of friction, from left to right for P1, P2, and P3, and from top to bottom for the BL5, GL2, ES3, and ES5 samples, with test conditions in the subtitle of each graph.

The tests followed a structured procedure to evaluate the effect of different surface conditions on the coefficient of friction. Three protocols were implemented. In protocol 1, 10 ml of water was sprayed at the start to create a uniformly wet surface. Protocol 2 added periodic wetting, with 3 ml of water applied every 120 seconds. Protocol 3 introduced a contamination layer by applying silicone grease before the initial water spray, followed by periodic wetting as in protocol 2. These protocols ensure a diverse and well-structured dataset, capturing distinct frictional behaviours under varying wet and contaminated conditions. See Fig. 3.

### 5.4 Measurement repeatability for reliable data acquisition

To ensure consistent repeatability of the frictional measurements, three specific protocols were established and employed to systematically compare the effects of varying wetting frequencies and pre-treatments on the frictional properties of the samples. Reproducibility is verified and ensured by applying consistent quantities of contaminants under identical test conditions, thereby guaranteeing reliable and repeatable results across experiments. Additionally, after each experiment, the disk and pin surfaces were cleaned using a commercial degreaser, followed by air-jet drying, to maintain consistent conditions for repeatability. Details of the friction measurements for the four samples BL5, GL2, ES3 and ES5, tested under the three protocols, are summarized in Tables 3, Table 4, Table 5.

**Table 3:** COF repeatability for used samples under protocol 1.

Test	BL5	GL2	ES3	ES5
Test 1	0.426	0.526	0.452	0.410
Test 2	0.444	0.500	0.459	0.435
Test 3	0.452	0. 537	0.406	0.429
Average	0.441	0.521	0.439	0.425
Std. Dev.	0.013	0.019	0.029	0.013

**Table 4:** COF repeatability for used samples under protocol 2.

Test	BL5	GL2	ES3	ES5
Test 1	0.467	0.537	0.437	0.434
Test 2	0.459	0.519	0.496	0.483
Test 3	0.445	0.487	0.449	0.449
Average	0.457	0.514	0.461	0.455
Std. Dev.	0.011	0.025	0.031	0.025

**Table 5:** COF repeatability for used samples under protocol 3.

Test	BL5	GL2	ES3	ES5
Test 1	0.195	0.274	0.253	0.323
Test 2	0.194	0.272	0.257	0.311
Test 3	0.194	0.266	0.262	0.320
Average	0.194	0.271	0.257	0.318
Std. Dev.	0.001	0.004	0.005	0.006

From Table 3, the standard deviations remain relatively low (ranging from 0.013 to 0.029) compared to the average Cof values, indicating a good level of measurement repeatability. Among the four samples, ES3 shows a slightly higher variation (Std. Dev. ≈ 0.029), but overall, these results suggest that Protocol 1 yields consistent friction measurements for each sample.

Table 4 shows that under protocol 2, the standard deviations (≈ 0.011 to 0.031) also indicate acceptable repeatability across the four samples. BL5 and ES5 both exhibit relatively small variability, whereas ES3 presents a slightly higher standard deviation (≈ 0.031). Still, the magnitudes of these deviations remain modest, implying that protocol 2 provides reliable measurements with only minor fluctuations in Cof readings.

Table 5 indicate that in protocol 3, the standard deviations range from ≈ 0.001 to 0.006, which is notably small relative to the mean Cof values. This suggests that the friction measurements under protocol 3 are highly repeatable. The results for BL5 are particularly consistent (Std. Dev. ≈ 0.001), indicating minimal experimental scatter. Overall, these figures confirm that protocol 3 offers very stable and reproducible Cof data for all four samples.

Overall, the three protocols demonstrate a satisfactory level of repeatability for the friction measurements across all tested samples. In each case, the standard deviations remain relatively small compared to the mean Cof values, indicating minimal variability between repeated tests. Although some protocols or sample types occasionally show slightly higher deviations, the degree of scatter is still modest. Consequently, these results confirm that the experimental setup and methods yield consistent, reliable measurements of friction properties under the specified conditions.

## Limitations

Not applicable.

## Ethics Statement

The authors confirm that they have read and adhere to the ethical requirements for publication in *Data in Brief*. This study does not involve human subjects, animal experiments, or data collected from social media platforms.

## CRediT Author Statement

**Bilel Jebali:** Data managing, Writing-Original draft preparation, Writing-Editing. **Manuela Gennesseaux:** Writing-Reviewing, Supervision. **Malal Kane:** Supervision, Project administration**.**

## Data Availability

zenodoDataset on contaminated steel/rubber contact: Effect of surface texture on friction coefficient (Original data)
